# Prognosis in autoimmune and infectious disease: new insights from genetics

**DOI:** 10.1038/cti.2014.8

**Published:** 2014-05-16

**Authors:** James C Lee, Kenneth G C Smith

**Affiliations:** 1 Cambridge Institute for Medical Research, Cambridge Biomedical Campus, Cambridge, UK; 2 Department of Medicine, University of Cambridge School of Clinical Medicine, Cambridge Biomedical Campus, Cambridge, UK

**Keywords:** autoimmunity, FOXO3, genetics, infection, prognosis

## Abstract

A well-recognised feature of autoimmune and infectious diseases is that their clinical course and eventual outcome can vary substantially between affected individuals. This variability in disease prognosis critically determines patient well-being, and yet is relatively poorly understood and largely understudied—with many investigators opting instead to study what causes disease development in the first place. Better understanding of what determines prognosis could provide unique insights into disease biology, potentially revealing new therapeutic targets, and will also be essential if prognosis-based ‘personalised medicine' is ever to become a reality. Here, we highlight the previously under-appreciated role that genetics has in determining prognosis in autoimmune and infectious disease, and the common role that FOXO3 has been shown to have as a modulator of inflammatory responses, and thereby of outcome, across several distinct diseases.

In 1918, Ronald Fisher proposed a genetic model in which human traits, including diseases, were determined, in the appropriate environmental context, by the inheritance of a sufficient number of risk alleles from across the genome.^1^ Nearly 90 years later the first genome-wide association studies (GWAS) confirmed his hypothesis and began a revolution in the field of complex disease genetics.^2^ By comparing the allele frequency at single-nucleotide polymorphisms (SNPs) located throughout the genome between cases and controls, GWAS facilitated the hypothesis-free discovery of genes that predispose to specific diseases, and led to unprecedented insights into the biology of disease development.^3^ When one considers how scant and hard-won our knowledge of complex disease genetics was before the advent of GWAS, these studies appear to have fulfilled their remit—to shed light upon the mechanisms of disease development. Nevertheless, despite this success, several criticisms of GWAS have been made. Chief among these is the fact that despite the apparent success, GWAS results have only explained a relatively small proportion of the total heritability of each disease.^3^ Work is now underway to try to identify the ‘missing heritability' through a variety of complementary methods, including whole-genome sequencing (to identify rare variants that may have larger effect sizes) and studies to examine interactions between a given gene and other genes (epistasis) and between genes and the environment. An unrelated but perhaps more practical criticism has also been made by several observers, who have highlighted the failure of GWAS results to be translated into patient benefit.^4, 5^ Although this criticism may seem surprising—for GWAS was not designed to inform treatment decisions or facilitate stratified medicine—it may reflect the growing recognition that disease development is only one aspect of disease biology, and that even a comprehensive understanding of this aspect may not explain important clinical phenomena—such as why two patients with the same diagnosis experience completely contrasting outcomes.

When faced with such prognostic heterogeneity, a major clinical challenge is to ensure that all patients receive appropriate therapy. This challenge is encountered in most medical specialities and—compounded by the inadequacy of ‘one-size-fits-all' approaches—has led to a widespread ambition to develop and deliver ‘personalised medicine'. By this approach, specific therapies would be targeted to individual patients in order to best manage the variability in disease behaviour. To realise this ambition, however, it will first be necessary to understand what determines disease prognosis.

Whether genetic variation might play a role in determining disease prognosis was largely unknown, for while disease susceptibility loci may not influence prognosis, this does not preclude the existence of other genetic variants that do. Indeed, given that GWAS identifies disease susceptibility loci through comparison of unstratified cases and controls, it would not be expected to reveal genetic variants that specifically associated with the outcome. This is because although the cases may share a common diagnosis, their clinical outcomes will be heterogeneous, such that a true prognostic variant may only be more common in a small subgroup of patients (for example, those with aggressive disease) and will also be reciprocally less common in another subgroup (for example, those with mild disease). We therefore hypothesised that to assess whether genetic variation influences disease course, a ‘within-cases' analysis would be necessary, in which the genetic profiles of patients with contrasting courses of disease could be directly compared (Figure 1). Here, we discuss our application of this approach to Crohn's disease (CD), a chronic and disabling form of inflammatory bowel disease, and the insights that a candidate gene study, recently published in *Cell,*^6^ have yielded both into the role of genetics in prognosis and also into the critical function of FOXO3 in regulating inflammation and thereby determining the outcome of autoimmune and infectious diseases.

## FOXO3, inflammatory responses and disease outcome

It was recently speculated that it should be possible to determine whether genetics influences prognosis in CD using existing GWAS data,^6^ even though this was published more than 5 years earlier.^2^ To do this, clinical data relating to the treatments that patients had required were first examined, and then subsequently used to identify two subgroups of patients who had experienced contrasting courses of CD. A direct comparison was then made between the genetic profiles of those patients who had required very little treatment over a prolonged follow-up period (‘mild disease') and those who had required multiple medical and surgical therapies because of recurrent disease flares and complications (‘aggressive disease').^6^ This comparison specifically focused on genes within the interleukin (IL)-2 and IL-7 signalling pathways, which have not been associated with CD development, but which are differentially expressed between patients with contrasting clinical outcomes in several autoimmune and inflammatory diseases, including CD.^7, 8^ This analysis led to the identification of a noncoding SNP within *FOXO3A* (rs12212067) at which the minor allele was shown to be consistently commoner in patients with a mild course of disease—with replication of this association being observed in several independent cohorts.^6^

*FOXO3A* encodes FOXO3, a member of the forkhead box O family of transcription factors, which also includes FOXO1 and FOXO4.^9^ These proteins are widely expressed and regulate diverse transcriptional programmes including cell-cycle control, apoptosis and metabolism.^9^ Much of our understanding of FOXO3 biology comes from animal models, in which Foxo1 and Foxo3 have been shown to have overlapping roles in regulatory T-cell development,^10, 11, 12^ while Foxo3 can additionally suppress inflammatory cytokine production in dendritic cells, resulting in reduced immune responses to infection^13^ and cancer.^14^

To fully characterise the functional effects of a human SNP is challenging, and doing so in patient cohorts risks confounding by disease activity and therapy, as well as the probability that other SNPs that exert similar effects elsewhere in a biological pathway are likely to be enriched within cohort being studied. To overcome this, we used a resource of genotyped healthy controls, who can be recalled to provide samples for functional experiments on the basis of their genotype (Cambridge BioResource, http://www.cambridgebioresource.org.uk) and which has previously supported some of the first functional studies of disease-associated SNPs in autoimmune disease.^15^

**Figure 2:**
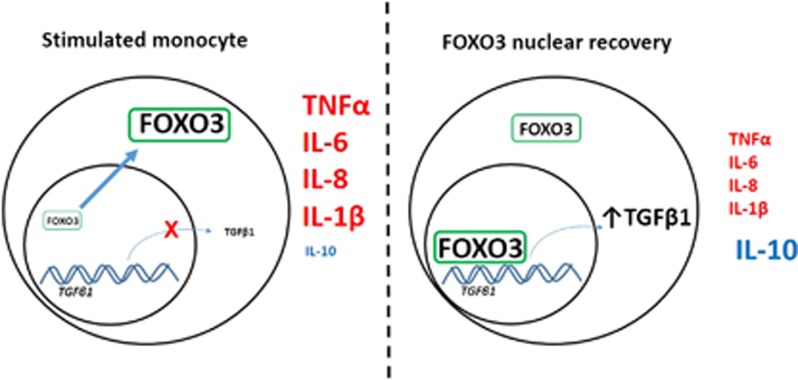
A FOXO3-driven pathway abrogates inflammatory responses in a transforming growth factor (TGF)-β1-dependent manner. Upon monocyte activation, FOXO3 is translocated out of the nucleus leading to the inactivation of its transcriptional programme. The re-instatement of this transcriptional programme is dependent upon transcription and *de novo* protein production, and leads, via a TGFβ1-dependent mechanism, to the abrogation of inflammatory responses by augmenting production of anti-inflammatory cytokines and suppressing that of pro-inflammatory cytokines. Minor allele carriage at rs12212067 (a SNP in *FOXO3A*) facilitates the earlier recovery of nuclear FOXO3 by increasing *FOXO3A* transcription during inflammatory responses and is associated with a milder course of Crohn's disease and rheumatoid arthritis but increased susceptibility to severe malaria.

These experiments revealed that minor allele carriage facilitates increased transcription of *FOXO3A* during monocyte activation, and that this in turn enables FOXO3's transcriptional programme to be re-instated earlier during an inflammatory response.^6^ Notably, this re-instatement was shown to initiate a transforming growth factor-β1-dependent pathway that reduced production of pro-inflammatory cytokines, including tumour necrosis factor (TNF)-α and IL-6, and increased production of the anti-inflammatory cytokine, IL-10 (Figure 2)—changes that are both consistent with a more indolent course of CD and which were mirrored in an *in vivo* model of colitis.^6^ On the basis of these observations, we then considered other diseases in which these cytokines are implicated—rheumatoid arthritis (RA), a chronic inflammatory polyarthritis in which TNFα contributes to pathogenesis^16^, and malaria, in which TNFα production contributes to an anti-microbial response that can be inhibited by IL-10.^17^ Outcome in both of these diseases can similarly vary between patients, and we further demonstrated that the minor allele at the *FOXO3A* SNP, which was associated with milder CD and lesser inflammatory responses, was also associated with a milder course of RA but with increased susceptibility to more severe malaria;^6^ consistent with the known role for these cytokines in each disease. Collectively, these results highlighted the clinically relevant insights that can be revealed through re-analysis of existing GWAS data, and revealed a previously unappreciated pathway by which common genetic variation in *FOXO3A* can manipulate inflammatory responses and thereby alter prognosis in distinct inflammatory and infectious diseases.^6^

## Complex disease genetics: moving beyond susceptibility

To date, studies of complex disease genetics have focused almost exclusively on disease development and susceptibility, with very little attention being paid to whether genetics contributes to other aspects of disease biology, such as prognosis/outcome. On the odd occasion where a role for genetics in disease prognosis has been considered, the methodology has often focused specifically on disease susceptibility variants, and the results have not delineated a clear role for them in driving disease outcome.^18, 19^ Moreover, some disease susceptibility variants are known to associate with specific subphenotypes of disease location and behaviour, which confound apparent associations with outcome. For example, the increased requirement for surgery in CD patients who carry *NOD2* variants^20^ is likely to be due to its specific association with complicated ileal disease^21^ rather than a more aggressive disease course *per se*. Given that the success of GWAS has been based on a strategy of comparing ‘affected' with ‘unaffected' individuals, we hypothesised that a potentially more rewarding approach to studying prognosis would be to directly compare the genetic profiles of patients with contrasting disease courses. Importantly, genetic variation in *FOXO3A* has never been associated with susceptibility to, or development of, any of these diseases. As such, this work has far-reaching implications for how we think about the genetics of complex disease, and introduces the idea that common genetic variation can determine the outcome of one or more diseases, without being associated with the disease itself.

To date, over 1000 GWAS studies have been performed to investigate the role of genetics in disease susceptibility,^22^ but very few have been re-examined since their initial publication. The concept that genetic variation can independently influence several aspects of disease biology has important ramifications for these studies, and may yet provide them with renewed utility if appropriate phenotypic data can be obtained to facilitate similar subphenotypic analyses. We have shown that this approach can reveal novel insights into disease biology, without the need to incur much additional expense, and can also uncover new drug targets that are relevant across several diseases. Whether such analyses could enable more accurate prediction of disease outcome or lead to the development of better targeted therapies is unknown, although both could potentially occur, and either would represent a major step towards the development of personalised medicine.

## Common pathways in disease prognosis

Modern medicine is largely based on the principle of diagnosis and the selection of treatment based on that diagnosis. In this way, clinicians are taught to subdivide diseases and place them accurately into distinct boxes in order to facilitate appropriate treatment. Although this approach is standard, it only accounts for the most obvious aspect of disease heterogeneity—namely, the differences in disease pathogenesis and the resulting clinical phenotype. Likewise, these subdivisions and resultant subspecialisations often mean that clinical research is similarly partitioned by disease label. Recently, genetics has highlighted common pathways that are involved in the pathogenesis of otherwise distinct diseases, such as IL-23 signalling in Psoriasis and CD^23^ and the role of *CTLA4* and regulatory T cells in type 1 diabetes and coeliac disease.^24^ Such insights have important implications for disease research but could also impact upon future treatment. The recent report that genetic variation in *FOXO3A* is associated with the prognosis of CD, RA and malaria^6^ provides yet further insights into the commonalities of disease biology, which are often overlooked by a reductionist disease classification system. For example, although most clinicians are aware that the course of an autoimmune or inflammatory disease can vary between patients, this is—to our knowledge—the first demonstration of a shared pathway that determines prognosis across otherwise distinct diseases. This finding would also support a model in which modulators of immune and inflammatory responses are largely responsible for determining the course of an inflammatory disease, irrespective of the original triggering events.

Moreover, we would further speculate that similar shared pathways—influencing, for example, T- or B-cell responses, regulatory T-cell function or neutrophil behaviour—may well be responsible for some of the other common features of autoimmune and inflammatory diseases. Collectively, therefore, we would envisage a situation in which many clinical aspects of otherwise distinct diseases are determined by common—rather than disease-specific—pathways. This hypothesis will clearly need testing, but if confirmed may lead to a new therapeutic approach, in which the goal of treatment may be to target those pathways that are responsible for aberrant behaviour in ongoing disease, such as a frequent rate of flare-ups, rather than on features associated with disease initiation. This approach could complement existing treatments, but would more directly target aspects of disease biology that are responsible for the greatest amount of disease morbidity. Accordingly, there would appear to be much to gain from greater study of those factors that are common between diseases aside from the most obvious phenotypic similarities, including disease course, relapse rates and/or response to specific therapies, as not only could this reveal more about disease biology, but it could also lead directly to better therapies in the future.

## Implications for improved patient care

Given the aforementioned criticisms of the clinical utility of GWAS associations, it is important to consider how observations leveraged through the study of disease prognosis might translate for patient benefit. For example, one might anticipate that such variants could be genotyped to enable clinicians to identify patients at risk of more aggressive disease and thereby facilitate tailored therapy. In the case of the prognostic SNP in *FOXO3A*, however, this is unlikely to be practical, for not only is the minor allele commoner in patients with a milder course of CD, but also the odds ratio is relatively low (0.6)—as is typical of SNPs identified using GWAS. This is in contrast to, for example, some of the genetic variants that occur within cancers and which are prognostically useful, such as *KRAS* mutations.^25^ Nonetheless, although the utility of single variants may be limited, it is possible that a broader panel of prognostic genetic variants—identified using a genome-wide approach—could be used to predict the likely course of disease so that treatments could be tailored. Several classification tools have been developed that use genome-wide SNP data to classify unknown subjects into cases/controls based on their genetic profiles and would seem ideally suited to this task.^26, 27^ The success of such an approach will thus depend mostly on the relative contribution that genetics makes to disease prognosis, and—as this is difficult to quantify—a trial-and-error approach may be necessary to determine whether clinically useful classification tools can be developed. Ultimately, we would envisage a situation in which several modalities might be used in conjunction to best predict the probable disease course. This may include the genotyping of a panel of prognostic SNPs, but is also likely to involve other methods, such as gene expression profiling—as we and others have described.^7, 8, 28^ Importantly, however, the value of studying the biology of disease prognosis does not hinge on the development of a successful classification tool. Nor, indeed, do we believe that attempts to generate clinically useful tools should be the primary objective of such work. As with GWAS, the primary goal should first be to better understand the biology underlying this aspect of disease—rather than to facilitate immediate translation—as this is likely to have more far-ranging benefits in the longer term. Indeed, although this may include the development of classification tools, other potential benefits such as the identification of novel therapeutic targets may be of equivalent or greater long-term value. Although this may appear speculative, this situation has already been exemplified in cardiovascular medicine, where genetic variants in the gene that encodes HMG Co-A reductase have been associated with low-density lipoprotein cholesterol levels.^29^ At first glance, the clinical utility of this observation may appear limited because the effect size (odds ratio) at these variants is small, and hence they cannot be used to accurately predict cholesterol levels. Nonetheless, pharmacological targeting of this enzyme using HMG Co-A reductase inhibitors (statins, which were actually developed before the genetic association) represents the most effective treatment for hypercholesterolaemia; highlighting the potential value of looking beyond genotypes into biological function. Similarly, although the *FOXO3A* SNP may have limited prognostic value in isolation, drugs that could target the pathways modulated by FOXO3 may be effective in a range of inflammatory diseases, potentially including some in which the genetics have not been examined.

It is noteworthy that this prognostic association and the critical role that FOXO3 has in modulating inflammatory responses were identified through the study of only 81 genes. It is unlikely that this is the only genetic determinant of prognosis, and thus a broader genome-wide analysis would be expected to reveal other genetic variants that contribute to disease prognosis. In doing so, such an analysis may lead to the identification of other pathways that can be targeted for therapeutic gain, or used to personalise treatment strategies. This, however, is not the only potential benefit of such an approach, as we would anticipate that it will also increase awareness of the role that genetics has in aspects of disease biology beyond susceptibility. In turn, we would hope that this will stimulate others to re-visit their own archived GWAS data to examine whether further insights can be similarly leveraged using clinically relevant ‘within cases' analysis.

## FOXO3, longevity and ‘inflammaging'

One unexpected consequence of this work is that the pathway that was identified appears to provide a unifying link between several factors that have been independently implicated in longevity. For example, it has often been reported that less intense inflammatory responses are associated with successful/healthy ageing, with centenarians displaying a milder age-related inflammatory phenotype,^30^ while interventions that increase lifespan in animals have been shown to reduce inflammatory markers.^31^ Such observations have already spawned interest in ‘inflammaging'^32^ and are supported by the observation that many age-related diseases have an inflammatory component (for example, atherosclerosis, dementia and so on). Indeed, the milder inflammatory responses observed in both humans and animals appear to be similar to those changes that are driven by the FOXO3-driven pathway; being characterised by less TNFα and more IL-10 production.^33^

Noncoding polymorphisms in *FOXO3A* have also been independently associated with healthy ageing in several populations.^34, 35, 36^ These genetic associations have been attributed to effects on insulin/insulin-like growth factor 1 signalling because of FOXO3's involvement in that pathway and its known link to ageing,^37^ although an additional effect acting through the control of inflammatory cytokine production is certainly imaginable.

## Conclusions

There is little doubt that in the last decade genetics has revolutionised our understanding of complex disease biology. However, it is equally important to appreciate that these achievements merely represent the end of the beginning. There are many aspects of disease biology that remain unexamined, and which—with appropriate study—could substantially advance our understanding of disease pathogenesis, and perhaps yield new therapeutic targets for better treatments. Recent work highlights the distinct contribution that genetics makes to prognosis in inflammatory and infectious disease, but this is also just one other aspect of disease behaviour. With similar structured approaches to GWAS data, it should be possible to leverage new insights into several other key areas of disease biology and, encouragingly, this should be achievable using existing data sets, which may have previously been thought to have little further value. We would anticipate that by exploiting the results of such studies, it may yet be possible to develop new prognostic and therapeutic strategies that are relevant across multiple diseases, and which could bring ‘personalised medicine' one step closer to reality.

## Figures and Tables

**Figure 1:**
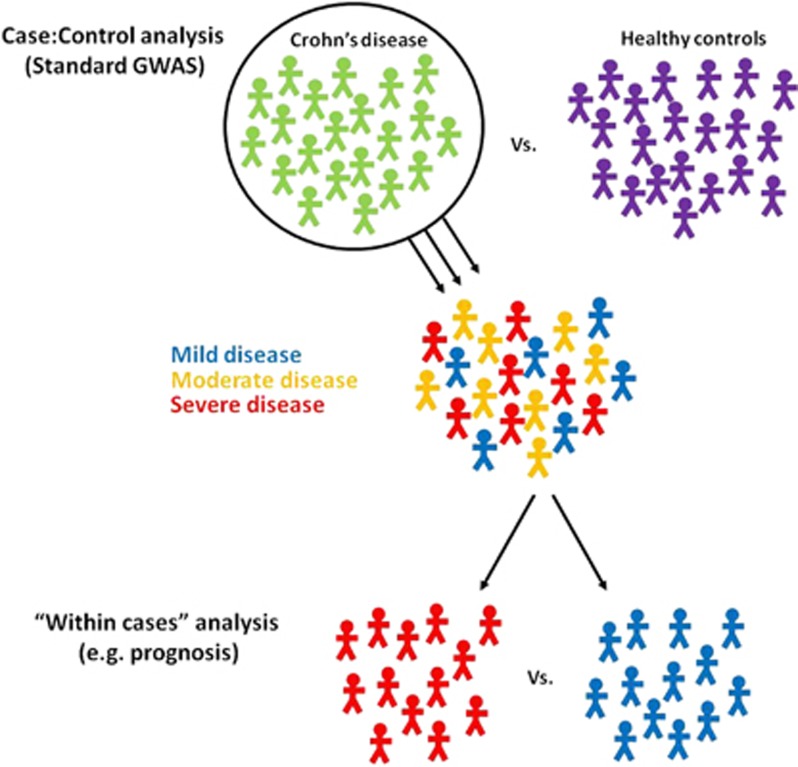
Comparison of patients with contrasting phenotypes within a GWAS cohort can leverage new insights into other aspects of disease biology. Standard GWAS analysis compares the allele frequency at single-nucleotide polymorphisms located throughout the genome between cases and controls, and thus facilitates the hypothesis-free discovery of genes that predispose to specific diseases. However, this approach will fail to account for important heterogeneity within the disease cohort. By exploiting allied phenotypic data, it is possible to examine the genetic contribution to such aspects of disease biology (including prognosis) by comparing the genetic profiles of patients with contrasting clinical phenotypes—a so-called ‘within-cases' analysis.

## References

[bib1] Fisher RA The correlation between relatives on the supposition of Mendelian inheritance Philos Trans R Soc Edin 1918 52 399 433

[bib2] Wellcome Trust Case Control Consortium Genome-wide association study of 14 000 cases of seven common diseases and 3000 shared controls Nature 2007 447 661 678 17554300 10.1038/nature05911 PMC2719288

[bib3] Jostins L Ripke S Weersma RK Duerr RH McGovern DP Hui KY Host-microbe interactions have shaped the genetic architecture of inflammatory bowel disease Nature 2012 491 119 124 23128233 10.1038/nature11582 PMC3491803

[bib4] McClellan J King MC Genetic heterogeneity in human disease Cell 2010 141 210 217 20403315 10.1016/j.cell.2010.03.032

[bib5] Crow TJ ‘The missing genes: what happened to the heritability of psychiatric disorders?' Mol Psychiatry 2011 16 362 364 21430674 10.1038/mp.2010.92

[bib6] Lee JC Espeli M Anderson CA Linterman MA Pocock JM Williams NJ Human SNP links differential outcomes in inflammatory and infectious disease to a FOXO3-regulated pathway Cell 2013 155 57 69 24035192 10.1016/j.cell.2013.08.034 PMC3790457

[bib7] McKinney EF Lyons PA Carr EJ Hollis JL Jayne DR Willcocks LC A CD8+ T cell transcription signature predicts prognosis in autoimmune disease Nat Med 2010 16 586 591581p following 591. 20400961 10.1038/nm.2130 PMC3504359

[bib8] Lee JC Lyons PA McKinney EF Sowerby JM Carr EJ Bredin F Gene expression profiling of CD8+ T cells predicts prognosis in patients with Crohn disease and ulcerative colitis J Clin Invest 2011 121 4170 4179 21946256 10.1172/JCI59255 PMC3196314

[bib9] Accili D Arden KC FoxOs at the crossroads of cellular metabolism, differentiation, and transformation Cell 2004 117 421 426 15137936 10.1016/s0092-8674(04)00452-0

[bib10] Ouyang W Beckett O Ma Q Paik JH DePinho RA Li MO Foxo proteins cooperatively control the differentiation of Foxp3+ regulatory T cells Nat Immunol 2010 11 618 627 20467422 10.1038/ni.1884

[bib11] Harada Y Elly C Ying G Paik JH DePinho RA Liu YC Transcription factors Foxo3a and Foxo1 couple the E3 ligase Cbl-b to the induction of Foxp3 expression in induced regulatory T cells J Exp Med 2010 207 1381 1391 20439537 10.1084/jem.20100004 PMC2901074

[bib12] Kerdiles YM Stone EL Beisner DR McGargill MA Ch'en IL Stockmann C Foxo transcription factors control regulatory T cell development and function Immunity 2010 33 890 904 21167754 10.1016/j.immuni.2010.12.002 PMC3034255

[bib13] Dejean AS Beisner DR Ch'en IL Kerdiles YM Babour A Arden KC Transcription factor Foxo3 controls the magnitude of T cell immune responses by modulating the function of dendritic cells Nat Immunol 2009 10 504 513 19363483 10.1038/ni.1729 PMC2712214

[bib14] Watkins SK Zhu Z Riboldi E Shafer-Weaver KA Stagliano KE Sklavos MM FOXO3 programs tumor-associated DCs to become tolerogenic in human and murine prostate cancer J Clin Invest 2011 121 1361 1372 21436588 10.1172/JCI44325 PMC3069771

[bib15] Dendrou CA Plagnol V Fung E Yang JH Downes K Cooper JD Cell-specific protein phenotypes for the autoimmune locus IL2RA using a genotype-selectable human bioresource Nat Genet 2009 41 1011 1015 19701192 10.1038/ng.434 PMC2749506

[bib16] Feldmann M Maini SR Role of cytokines in rheumatoid arthritis: an education in pathophysiology and therapeutics Immunol Rev 2008 223 7 19 18613827 10.1111/j.1600-065X.2008.00626.x

[bib17] Hugosson E Montgomery SM Premji Z Troye-Blomberg M Bjorkman A Higher IL-10 levels are associated with less effective clearance of *Plasmodium falciparum* parasites Parasite Immunol 2004 26 111 117 15279621 10.1111/j.0141-9838.2004.00678.x

[bib18] Weersma RK Stokkers PC van Bodegraven AA van Hogezand RA Verspaget HW de Jong DJ*, et al.* Molecular prediction of disease risk and severity in a large Dutch Crohn's disease cohort Gut 2009 58 388 395 18824555 10.1136/gut.2007.144865

[bib19] Cleynen I Mahachie John JM Henckaerts L Van Moerkercke W Rutgeerts P Van Steen K Molecular reclassification of Crohn's disease by cluster analysis of genetic variants PLoS One 2010 5 e12952 20886065 10.1371/journal.pone.0012952 PMC2944846

[bib20] Annese V Lombardi G Perri F D'Inca R Ardizzone S Riegler G Variants of CARD15 are associated with an aggressive clinical course of Crohn's disease—an IG-IBD study Am J Gastroenterol 2005 100 84 92 15654786 10.1111/j.1572-0241.2005.40705.x

[bib21] Abreu MT Taylor KD Lin YC Hang T Gaiennie J Landers CJ Mutations in NOD2 are associated with fibrostenosing disease in patients with Crohn's disease Gastroenterology 2002 123 679 688 12198692 10.1053/gast.2002.35393

[bib22] Hindorff LA MacArthur J Morales J Junkins HA Hall PN Klemm AKA Catalog of Published Genome-Wide Association Studies. Available at: http://www.genome.gov/gwastudies 2013Accessed December 2013..

[bib23] Ellinghaus D Ellinghaus E Nair RP Stuart PE Esko T Metspalu A Combined analysis of genome-wide association studies for Crohn disease and psoriasis identifies seven shared susceptibility loci Am J Hum Genet 2012 90 636 647 22482804 10.1016/j.ajhg.2012.02.020 PMC3322238

[bib24] Smyth DJ Plagnol V Walker NM Cooper JD Downes K Yang JH Shared and distinct genetic variants in type 1 diabetes and celiac disease N Engl J Med 2008 359 2767 2777 19073967 10.1056/NEJMoa0807917 PMC2840835

[bib25] Lievre A Bachet JB Le Corre D Boige V Landi B Emile JF KRAS mutation status is predictive of response to cetuximab therapy in colorectal cancer Cancer Res 2006 66 3992 3995 16618717 10.1158/0008-5472.CAN-06-0191

[bib26] Sambo F Trifoglio E Di Camillo B Toffolo GM Cobelli C Bag of Naive Bayes: biomarker selection and classification from genome-wide SNP data BMC Bioinformatics 2012 13 (Suppl 14 S2 23095127 10.1186/1471-2105-13-S14-S2 PMC3439675

[bib27] de Los Campos G Vazquez AI Fernando R Klimentidis YC Sorensen D Prediction of complex human traits using the genomic best linear unbiased predictor PLoS Genet 2013 9 e1003608 23874214 10.1371/journal.pgen.1003608 PMC3708840

[bib28] Arijs I Li K Toedter G Quintens R Van Lommel L Van Steen K Mucosal gene signatures to predict response to infliximab in patients with ulcerative colitis Gut 2009 58 1612 1619 19700435 10.1136/gut.2009.178665

[bib29] Kathiresan S Melander O Anevski D Guiducci C Burtt NP Roos C Polymorphisms associated with cholesterol and risk of cardiovascular events N Engl J Med 2008 358 1240 1249 18354102 10.1056/NEJMoa0706728

[bib30] Bruunsgaard H Andersen-Ranberg K Hjelmborg J Pedersen BK Jeune B Elevated levels of tumor necrosis factor alpha and mortality in centenarians Am J Med 2003 115 278 283 12967692 10.1016/s0002-9343(03)00329-2

[bib31] Horrillo D Sierra J Arribas C Garcia-San Frutos M Carrascosa JM Lauzurica N Age-associated development of inflammation in Wistar rats: Effects of caloric restriction Arch Physiol Biochem 2011 117 140 150 21635187 10.3109/13813455.2011.577435

[bib32] Franceschi C Capri M Monti D Giunta S Olivieri F Sevini F Inflammaging and anti-inflammaging: a systemic perspective on aging and longevity emerged from studies in humans Mech Ageing Dev 2007 128 92 105 17116321 10.1016/j.mad.2006.11.016

[bib33] Licastro F Candore G Lio D Porcellini E Colonna-Romano G Franceschi C Innate immunity and inflammation in ageing: a key for understanding age-related diseases Immun Ageing 2005 2 8 15904534 10.1186/1742-4933-2-8 PMC1166571

[bib34] Flachsbart F Caliebe A Kleindorp R Blanche H von Eller-Eberstein H Nikolaus S Association of FOXO3A variation with human longevity confirmed in German centenarians Proc Natl Acad Sci USA 2009 106 2700 2705 19196970 10.1073/pnas.0809594106 PMC2650329

[bib35] Anselmi CV Malovini A Roncarati R Novelli V Villa F Condorelli G Association of the FOXO3A locus with extreme longevity in a southern Italian centenarian study Rejuvenation Res 2009 12 95 104 19415983 10.1089/rej.2008.0827

[bib36] Soerensen M Dato S Christensen K McGue M Stevnsner T Bohr VA Replication of an association of variation in the FOXO3A gene with human longevity using both case-control and longitudinal data Aging Cell 2010 9 1010 1017 20849522 10.1111/j.1474-9726.2010.00627.x PMC2992870

[bib37] Ziv E Hu D Genetic variation in insulin/IGF-1 signaling pathways and longevity Ageing Res Rev 2011 10 201 204 20868776 10.1016/j.arr.2010.09.002

